# Establishment of a National Inventory of Dangerous Pathogens in the Republic of Uganda

**DOI:** 10.1089/hs.2018.0112

**Published:** 2019-06-14

**Authors:** Sabrina Brizee, Musa Kwehangana, Collins Mwesigwa, Diederik A. Bleijs, Harold H. J. L. van den Berg, Evelien Kampert, Milton Wetaka Makoba, Atek Kagirita, Issa Makumbi, Francis Kakooza, Maxwell Otim Onapa, Mark W. J. van Passel

**Affiliations:** Sabrina Brizee, Diederik A. Bleijs, Harold H. J. L. van den Berg, Evelien Kampert, and Mark W. J. van Passel are with the National Institute for Public Health and the Environment, Bilthoven, The Netherlands. Musa Kwehangana, Collins Mwesigwa, and Maxwell Otim Onapa are with the Uganda National Council for Science and Technology, Kampala, Uganda. Milton Wetaka Makoba and Issa Makumbi are with the Emergency Operations Centre, Ministry of Health, Kampala, Uganda. Atek Kagirita is with the Central Public Health Laboratories, Ministry of Health, Kampala, Uganda. Francis Kakooza is with the Infectious Disease Institute, Makarere University, Kampala, Uganda.

**Keywords:** Biological Weapons Convention (BWC), Legislative issues, National strategy/policy, Surveillance

## Abstract

One of the challenges of global biosecurity is to protect and control dangerous pathogens from unauthorized access and intentional release. A practical and feasible option to protect life science institutes against theft and sabotage, and secure their biological materials against misuse, is to establish a national electronic database with a comprehensive overview of the locations of all controlled dangerous pathogens in a country. This national database could be used as an instrument to secure and account for dangerous pathogens in a country, but it could also assist in establishing a biosecurity assessing and monitoring system for laboratories that work with these controlled biological agents. The Republic of Uganda is one of the first countries, prompted by the World Health Organization's (WHO's) Joint External Evaluation (JEE), to implement a national electronic database that assembles information collected from relevant Ugandan laboratories. This Ugandan Inventory of Dangerous Pathogens is different from an institute-specific pathogen inventory system, as it is intended to store the information collected from laboratories in the country working with dangerous pathogens in 1 centralized secure location. The Uganda National Council for Science and Technology (UNCST) has coordinated the implementation of the Ugandan national inventory. The inventory was recognized by the WHO JEE as contributing to Uganda's developed capacities regarding biosafety and biosecurity. This article describes the steps in implementing Uganda's National Inventory of Dangerous Pathogens. In addition, it presents a straightforward approach that can be adapted by other countries that aim to enhance their biosecurity capacities.

## Abstract 2

The Republic of Uganda is one of the first countries to implement a national electronic database that assembles information collected from relevant Ugandan laboratories. This Ugandan Inventory of Dangerous Pathogens is different from an institute-specific pathogen inventory system, as it is intended to store the information collected from laboratories in the country working with dangerous pathogens in a centralized secure location.

The multilateral Biological Weapons Convention (BWC) was established in 1972 to free the world of the development, production, and stockpiling of biological weapons of mass destruction.^1^ The current 180 signatory countries that have ratified the BWC have pledged to implement measures to improve international cooperation in the field of peaceful biological activities. In addition, United Nations Security Council Resolution 1540 (UNSCR 1540) of 2004 states specifically that countries should “develop and maintain appropriate effective measures to account for and secure such items (i.e. nuclear, chemical or biological weapons) in production, use, storage or transport.”^2^

Although signatory parties are called on to take appropriate measures to secure and account for dangerous pathogens against misuse, governments first must determine which dangerous pathogens are in fact present in their country. This helps governments assess biosecurity needs in order to respond to infectious disease outbreaks and prevent deliberate misuse of dangerous pathogens. In addition, the data from the national inventory can be used as the basis of establishing appropriate biosecurity assessment and monitoring systems.

The importance of having a national inventory in place is shown by the fact that several incidents of unintentional spread of dangerous pathogens,^3^ or the potential risk thereof,^3-6^ have been noted since the establishment of the BWC. Several instances of the intentional misuse of pathogenic microbes have been documented,^7-9^ the most infamous being the anthrax letters in 2001.

Fortunately, the world has since seen no large-scale calamities by biological warfare, bioterrorism, or biocrime.^10^ Although a national inventory may not contribute to the prevention of a catastrophic biological event, the information in this database constitutes a valuable resource for enhancing national capacities to prevent, detect, and respond to infectious diseases.

In 2014, the Global Health Security Agenda (GHSA) was established as a multilateral and multisectoral partnership to combat infectious diseases worldwide. This initiative was divided into 11 action packages to facilitate progress toward the common GHSA goals. One of these action packages is biosafety and biosecurity,^11^ which aims to initiate the “implementation of a comprehensive, sustainable and legally embedded national oversight program for biosafety and biosecurity, including the safe and secure use, storage, disposal, and containment of pathogens found in laboratories and a minimal number of holdings across the country, including research, diagnostic and biotechnology facilities.”

Uganda, one of the GHSA pilot countries, conducted a self-assessment in 2010 of the scope of biosecurity in the country with a view to making biosecurity policy recommendations.^12^ The self-assessment, which was undertaken by UNCST and the Uganda National Academy of Sciences (UNAS), produced a number of recommendations. The first was that Uganda should have an accurate inventory of relevant laboratories and the infectious agents in the country. This recommendation was subsequently included in Uganda's GHSA 5-year roadmap of 2015.

Prior to the 2014 West Africa Ebola outbreak, Uganda was the country with the largest epidemic of viral hemorrhagic virus infections.^13^ Besides Ebola and Marburg viruses, many dangerous pathogens are endemic in Uganda, such as the plague, anthrax, and cholera,^14,15^ and it is a hotspot for other emerging and reemerging pathogens. The Ugandan Ministry of Health established a public health emergency operations center (PHEOC) to protect the public from these newly emerging or reemerging infectious diseases. The center allows Uganda to respond to outbreaks in a more coherent, effective, and efficient manner. In 2014, the ministry of health, the UNCST, and the Netherlands National Institute for Public Health and the Environment (RIVM) collaborated to establish a National Inventory of Dangerous Pathogens tailored to Uganda.

Uganda's inventory of dangerous pathogens is a secure, centralized electronic database intended to store information collected from those institutes working with dangerous pathogens. This information could assist the government of Uganda in identifying national biosecurity needs, an initial step in developing a meaningful biosecurity policy and setting up systems to monitor biosecurity performance of laboratories. For example, institutes that work with dangerous pathogens could be required to implement specific security measures to mitigate risks of sabotage, loss, theft, misuse, and intentional release of biological agents. A national inventory could also help in preparing emergency response plans and by identifying national reference laboratories for outbreak specimens. Lastly, a national inventory of dangerous pathogens is in compliance with the BWC framework, the UNSCR 1540 resolution, the GHSA biosafety and biosecurity action package aims, and the Ugandan national biosecurity recommendations.^12^

The UNCST carried out the implementation process of the Ugandan national inventory and has been recognized by the World Health Organization (WHO) Joint External Evaluation (JEE) as contributing to Uganda's developed capacities regarding biosafety and biosecurity. Uganda was one of the first to implement a national inventory of dangerous pathogens, assembling information collected from all relevant institutes. This paper highlights Uganda's effort in establishing a National Inventory of Dangerous Pathogens by focusing on the required phases and related steps for successful implementation. In addition, it presents a straightforward approach that can be adapted by other countries that aim to enhance their biosecurity capacities.

## Methods

### Software

The RIVM has provided secure software to the UNCST to create and manage a national inventory of dangerous pathogens in Uganda. The national inventory is survey-driven. Data can be collected through a spreadsheet, then imported and securely stored into the electronic database. The National Inventory of Dangerous Pathogens in Uganda stores information on all relevant institutes, their geographic location, the select agents these laboratories are storing and handling, their safety classification, and the contact details of the responsible biosafety officer. The database also provides options for adding additional information manually. The information stored in the databases should be considered sensitive information and protected with a high level of security.

### Stakeholder Consultation

Before the implementation process of the national inventory, the RIVM and the public health emergency operations center conducted a stakeholder consultation, which included 4 site visits with key Ugandan institutes, assigned by the public health emergency operations center, the Infectious Disease Institute (IDI) of Makarere University, the National Public Health Laboratory Services (NPHLS), the Uganda Virus Research Institute (UVRI), and the National Animal Disease and Diagnostic Epidemiology Centre (NADDEC). The main purpose of this stakeholder consultation was to recommend to the UNCST how best to implement a national inventory of dangerous pathogens. These recommendations would ensure that the database aligns with the specific biosecurity needs of the country.

These expert opinions resulted in a set of recommendations. First, the national inventory would store information from all selected institutes, with a short note that the stakeholders' list of the national inventory would need to be revised periodically. Next, selected institutes would be required to report only biological agents on the US Federal Select Agents List,^16^ the 67 biological agents that have been determined to pose a severe threat to human, plant, and animal health. Laboratories should report any of those agents that have been stored longer than 2 weeks. Third, access to the National Inventory of Dangerous Pathogens would be granted only to authorized individuals from the UNCST and would be reported to the responsible authorities.

### Implementation Process

The implementation of a National Inventory of Dangerous Pathogens can be divided into 3 stages: preparation, implementation, and maintenance. In the preparatory phase, the government of Uganda committed to the establishment of a National Inventory of Dangerous Pathogens and assigned responsibilities in the government.

With a designated government focal point in Uganda, the implementation phase was initiated, and a communication plan was set up for contacting the appropriate institutes for the relevant data. The list of these institutes was compiled with the help of IDI, UVRI, UNCST, NPHLS, and NADDEC, in addition to the Biosafety and Biosecurity Association Uganda, and included approximately 40 institutes being requested to provide relevant information. The decision of the prioritized pathogen list to be included in the inventory was determined to be the US Federal Select Agents List, and the information was gathered and inserted into the dedicated software. In the maintenance phase, the focal point is responsible for informing the appropriate Ugandan authorities (ie, the ministry of health, as part of the team for the JEE, and the Uganda representative at the BWC) about the number and location of institutes storing dangerous pathogens, as well as the variety of dangerous pathogens present in the Republic of Uganda and plans for annual updates of the inventory.

In all of these stages, communication, ownership, and data-collection activities lie with the Uganda focal point, and no sensitive information was shared with or handled by anyone not approved by the focal point. The database will be owned and controlled by the government of Uganda. Although institutional data on working with high-risk pathogens may not be sensitive information, the consolidated national data could be considered sensitive information and should therefore be stored safely and securely according to Ugandan procedures and relevant official information confidentiality laws.

The public health emergency operations center of the ministry of health in Uganda initially served as the focal point for organizing, coordinating, supporting, and managing all aspects of public health emergency response efforts. Subsequently, ownership of the national point was handed over from the ministry of health to the UNCST, which would carry out the laboratory mapping activities, host and maintain inventory, and report to the relevant ministries. Two professionals from the UNCST were active on this project, which included an IT specialist and a biosafety and biosecurity expert, which is sufficient for the maintenance phase during the annual updates. Support from the Netherlands included a program coordinator, a program officer, and an additional subject matter expert. There were 6 in-person meetings in Uganda: 1 meeting with the ministry of health director general for commitment, 1 meeting for determining the responsible government entity, 1 dedicated to site visits to the 4 large Ugandan institutes working on human and zoonotic pathogens, 1 meeting dedicated to IT infrastructure, 1 dedicated to communication strategies, and 1 evaluative meeting.

## Results and Discussion

### Finalizing the National Inventory

The implementation process took approximately 1 year and included the government and institutional commitment process, identifying all relevant laboratories in the country, allocating responsibilities regarding the implementation process, assigning access rights to the information, setting up the communication plan, arranging the dedicated and secure IT infrastructure at a central location, and finally collecting, registering, and processing the requested electronic information from Ugandan institutes.

The National Inventory of Dangerous Pathogens in Uganda stores electronic data on approximately 40 institutes in the country and their geographic locations, the select agents these laboratories are storing and their safety classification, and the contact details of the responsible biosafety officer. It is important to note that not all institutes were necessarily storing dangerous pathogens, but it was considered relevant to store information on all institutes and include these in subsequent annual updates.

Finally, this resulted in an operational and accurate Ugandan National Inventory of Dangerous Pathogens. Since a National Inventory of Dangerous Pathogens contains sensitive information, this case study therefore provides no concrete information on the contents of this inventory.

In 2017, the WHO JEE scored the biosafety and biosecurity capacities of the Republic of Uganda as “developed capacity” (score 3 out of 5),^17^ which was a substantial increase in the score from the 2015 GHSA pilot assessment in Uganda. Although a number of recommendations in the area of biosafety and biosecurity still must be fulfilled, the initial inventory was specifically mentioned as one of the strengths and best practices in the whole of government biosafety and biosecurity system.^17^ By including the National Inventory in the JEE process, the government of Uganda showed continued commitment to this activity.

### Challenges Encountered

Several challenges were encountered during the various stages of implementation. For example, in the preparatory phase, there was initially some confusion about the aims of the National Inventory of Dangerous Pathogens, which was occasionally considered to be an actual (physical) specimen collection. This required an elaboration on the concept of the inventory regarding its purpose and the international regulations and frameworks, emphasizing that the inventory is an electronic resource only. Some institutes were initially reluctant to disclose information on high-risk pathogens. This highlighted the need to raise awareness about the purpose of the inventory, its relevance to national biosecurity, and what is expected from institutes concerning data sharing. Communication on the commitment by the government of Uganda contributed to more compliance. One recommendation was that prior to the first official communication, a workshop with representatives of all institutes would have been helpful in raising awareness and providing tailored information.

An additional challenge is that the BWC and other international treaties rarely specify what constitutes dangerous pathogens, and therefore no protocols exist on which pathogens should be monitored and controlled. Decisions on such issues can be challenging when a large number of stakeholders are involved. For this reason, it was decided to include organisms from the US Federal Select Agent List, with the possibility to design and implement a national prioritized pathogen list in the Ugandan context at a later stage. Although the various international regulations concerning biosecurity provide little guidance on how precisely to set up a practical system to account for and secure biological agents and toxins, there is some level of flexibility regarding the relevant elements to be included in the electronic database. Still, it was important to decide early in the process what elements pertinent to Uganda should be included.

## Conclusions

In 2016, the government of Uganda successfully implemented a National Inventory of Dangerous Pathogens. This activity has been formally recognized in the WHO JEE of 2017 as contributing to the Ugandan operational capacity regarding biosafety and biosecurity. In 2018, the UNCST organized a follow-up activity: a national stakeholders meeting to discuss the national biosecurity framework and to simultaneously update the National Inventory of Dangerous Pathogens, thereby ensuring sustainable implementation.

A National Inventory of Dangerous Pathogens could contribute to several national biosafety and biosecurity issues simultaneously. First, an accurate inventory is in line with international regulations (eg, the BWC) concerning nonproliferation commitments. Second, a national inventory of dangerous pathogens could provide meaningful information for establishing a national policy framework on biosafety and biosecurity, based on the actual occurrence of pathogens in-country. Third, emergency response plans for both intentional and accidental biological calamities in laboratories can be more appropriately tailored when the government, and by extension first responders, has access to accurate information concerning the presence of dangerous pathogens in facilities. Additionally, an existing national inventory could aid in the preparations of emergency response plans—for example, by identifying national reference laboratories for infectious disease outbreaks.

Numerous WHO JEEs have been conducted, and the biosafety and biosecurity recommendation frequently noted is the need to set up and implement a national pathogen inventory system.^18,19^ The fact that numerous countries still do not have a meaningful system in place to account for dangerous pathogens indicates that such inventories constitute a significant biosecurity gap worldwide. This need also coincides with the fundamental biosecurity priority of the Global Partnership against the Spread of Weapons and Materials of Mass Destruction: that states should secure and account for materials that represent biological proliferation risks.

Currently, a guidance document on how to implement a national inventory of dangerous pathogens for other interested countries is still lacking. However, as the Uganda experience demonstrates, the establishment of such an inventory is feasible and practical, but requires tailored, country-specific protocols conscientiously implemented.

## References

[1] United Nations Office for Disarmament Affairs. Biological weapons. The Biological Weapons Convention. https://www.un.org/disarmament/wmd/bio/ Accessed 415, 2019

[2] 1540 Committee. Security Council Committee established pursuant to resolution 1540 (2004). https://www.un.org/en/sc/1540/ Accessed 415, 2019

[3] Wade N Death at Sverdlovsk: a critical diagnosis. Science 1980;209(4464):1501-1502 7433972 10.1126/science.7433972

[4] Reardon S ‘Forgotten' NIH smallpox virus languishes on death row. Nature 2014;514(7524):544 25355337 10.1038/514544a

[5] Rodrigues AL, Silva SK, Pinto BL, Silva JB, Tupinambas U Outbreak of laboratory-acquired Brucella abortus in Brazil: a case report. Rev Soc Bras Med Trop 2013;46(6):791-794 24474027 10.1590/0037-8682-0160-2013

[6] Weiss S, Yitzhaki S, Shapira SC Lessons to be learned from recent biosafety incidents in the United States. Isr Med Assoc J 2015;17(5):269-273 26137650

[7] Keim P, Smith KL, Keys C, Takahashi H, Kurata T, Kaufmann A Molecular investigation of the Aum Shinrikyo anthrax release in Kameido, Japan. J Clin Microbiol 2001;39(12):4566-4567 11724885 10.1128/JCM.39.12.4566-4567.2001 PMC88589

[8] McCarthy M Anthrax in USA—attacks “deadly but treatable.” Lancet 2001;358(9292):1520 11705575 10.1016/S0140-6736(01)06625-9

[9] Torok TJ, Tauxe RV, Wise RP, et al. A large community outbreak of salmonellosis caused by intentional contamination of restaurant salad bars. JAMA 1997;278(5):389-395 9244330 10.1001/jama.1997.03550050051033

[10] Jansen HJ, Breeveld FJ, Stijnis C, Grobusch MP Biological warfare, bioterrorism, and biocrime. Clin Microbiol Infect 2014;20(6):488-496 24890710 10.1111/1469-0691.12699 PMC7129974

[11] Centers for Disease Control and Prevention. Global Health Security Agenda: action packages. Last reviewed January 21, 2016. https://www.cdc.gov/globalhealth/security/actionpackages/ Accessed 415, 2019

[12] Committee on Assessing the Current State of Knowledge Pertaining to the Meaning and Scope of Bio-safety and Bio-Security in the Context of Uganda. The Scope of Biosafety and Biosecurity in Uganda: Policy Recommendations for the Control of Associated Risks. Kampala: Uganda National Academy of Sciences; 2010 http://www.vertic.org/media/National%20Legislation/Uganda/UG_Scope_Biosafety_Biosecurity_Uganda.pdf

[13] Nyakarahuka L, Kankya C, Krontveit R, et al. How severe and prevalent are Ebola and Marburg viruses? A systematic review and meta-analysis of the case fatality rates and seroprevalence. BMC Infect Dis 2016;16(1):708 27887599 10.1186/s12879-016-2045-6 PMC5124280

[14] Bwire G, Malimbo M, Makumbi I, et al. Cholera surveillance in Uganda: an analysis of notifications for the years 2007-2011. J Infect Dis 2013;208(Suppl 1):S78-S85 24101649 10.1093/infdis/jit203

[15] Sekamatte M, Krishnasamy V, Bulage L, et al. Multisectoral prioritization of zoonotic diseases in Uganda, 2017: a One Health perspective. PLoS One 2018;13(5):e0196799 29715287 10.1371/journal.pone.0196799 PMC5929520

[16] Centers for Disease Control and Prevention. Federal select agents program. https://www.selectagents.gov/ Accessed 415, 2019

[17] World Health Organization. Joint External Evaluation of IHR Core Capacities of the Republic of Uganda. 2017 www.who.int/ihr/publications/WHO-WHE-CPI-REP-2017.49/en/ Accessed 415, 2019

[18] World Health Organization. Joint External Evaluation of IHR Core Capacities of the Federal Democratic Republic of Ethiopia. 2016 www.who.int/ihr/publications/WHO-HSE-GCR-2016.24/en/ Accessed 415, 2019

[19] World Health Organization. Joint External Evaluation of IHR Core Capacities of the Republic of Kenya. 2017 www.who.int/ihr/publications/WHO-WHE-CPI-REP-2017.44/en/ Accessed 415, 2019

